# Plant–Aphid Interactions in Cereal Crops: Wheat Plant Defense and Aphid Saliva-Mediated Coevolutionary Arms Race

**DOI:** 10.3390/insects17070672

**Published:** 2026-06-27

**Authors:** Xiaobei Liu, Qian Wang, Yong Liu, Yong Zhang, Frédéric Francis, Julian Chen

**Affiliations:** 1 State Key Laboratory for Biology of Plant Diseases and Insect Pests, Institute of Plant Protection, Chinese Academy of Agricultural Sciences, Beijing 100193, China; xiaobeiliu7@163.com; 2 Functional and Evolutionary Entomology, Gembloux Agro-BioTech, University of Liège, 5030 Gembloux, Belgium; 3 College of Plant Protection, Hebei Agricultural University, Baoding 071000, China; 4 College of Plant Protection, Shandong Agricultural University, Taian 271018, China; 5 Center for Biosafety, Chinese Academy of Inspection and Quarantine, Sanya 572024, China

**Keywords:** wheat aphids, plant defense, non-host resistance, resistance genes, salivary proteins

## Abstract

Wheat aphids are among the most destructive pests of wheat, posing a serious threat to wheat production and global food security. The development of aphid-resistant wheat varieties is widely regarded as one of the most economical and sustainable strategies for aphid management. A comprehensive understanding of the plant defense mechanisms is fundamental to the breeding of resistant wheat germplasm. Recent studies have revealed that aphid salivary proteins play critical roles in manipulating host defenses, whereas wheat has evolved multiple layers of defense to perceive and respond to aphid attack. In this review, we summarize current advances in wheat–aphid interactions, focusing on the roles of aphid salivary effectors, aphid-induced immune signaling, physical and chemical defense barriers, herbivore-induced plant volatiles (HIPVs), resistance genes, and non-host resistance mechanisms. By integrating aphid effector biology with host immunity and resistance mechanisms, this review provides a conceptual framework for understanding wheat–aphid interactions and for developing durable and environmentally sustainable resistance strategies.

## Simple Summary

Wheat aphids are among the most damaging pests of wheat worldwide. Although resistant cultivars provide an environmentally friendly and sustainable method of aphid control, the molecular mechanisms underlying durable resistance remain incompletely understood. In recent years, increasing evidence has demonstrated that aphid salivary proteins serve as critical mediators in the interaction between wheat and aphids. This review synthesizes current advances in our understanding of how wheat perceives aphid attack and activates multiple defense layers, including signaling pathways, physical and chemical defenses, resistance genes, and non-host resistance mechanisms. We further discuss key knowledge gaps and emerging research opportunities that may facilitate the development of durable aphid-resistant wheat varieties and more sustainable pest management strategies.

## 1. Introduction

Wheat is an important crop widely cultivated around the world and plays a critical role in global food security [1]. Wheat aphids are among the most destructive pests of wheat, as they feed directly on phloem sap [2]. Characterized by their small body size, short generation time, high reproductive capacity, and rapid population growth, they pose a serious threat to wheat production throughout all stages of crop development [2]. The main species of wheat aphids include *Sitobion avenae*, *Rhopalosiphum padi*, *Schizaphis graminum*, *Metopolophium dirhodum*, and *Diuraphis noxia*. Currently, chemical pesticides remain heavily relied upon for the management of wheat aphids, leading to a series of problems including pest resistance, adverse effects on beneficial insects and environmental pollution [3]. Therefore, it is urgent to reduce the use of chemical pesticides and enhance the ecological benefits of wheat-based agroecosystems. Breeding and utilizing aphid-resistant wheat varieties is one of the most cost-effective strategies for sustainable aphid management. However, there is a lack of aphid-resistant germplasm among existing wheat varieties.

In the “arms race” of coevolution between plants and herbivorous insects, plants have evolved diverse direct and indirect defense mechanisms to counter insect feeding, thereby enhancing plant resistance to insect pests [4]. During plant defense responses, signaling molecules such as phytohormones play crucial roles in regulating defense signaling networks [5]. Plant resistance can be categorized into non-host and host resistance, of which the latter has been more extensively studied. Host resistance to aphids is generally exhibited through antixenosis, antibiosis, and tolerance, which are mediated by a combination of constitutive and inducible defense mechanisms [6].

Considerable progress has been made in understanding wheat resistance to aphids, particularly in the areas of plant defense signaling, resistance genes, and aphid-induced defense responses. However, the molecular mechanisms governing wheat–aphid interactions remain incompletely understood. In recent years, aphid salivary proteins have emerged as key determinants of plant–aphid interactions, functioning either as elicitors that activate host defenses or as effectors that suppress plant immunity [7,8]. However, current identification of aphid salivary effectors and elicitors is limited to functional studies of individual salivary proteins. Their molecular targets in wheat have not been explored in depth, and the signaling network connecting aphid perception and effective resistance response has not been fully elucidated. In addition, only a limited number of aphid-resistance genes have been characterized and utilized in breeding programs, which has constrained the development of wheat cultivars with durable and broad-spectrum resistance. Meanwhile, non-host resistance, which is often more durable and broad-spectrum than host resistance [9,10], remains relatively underexplored in wheat–aphid systems despite its considerable potential for resistance breeding.

To address these challenges, this review summarizes current knowledge of wheat–aphid interactions from the perspective of the molecular arms race between aphids and their host plants. We focus on the roles of aphid salivary proteins in modulating plant defenses and discuss the multiple layers of wheat resistance, including defense signaling pathways, physical and chemical barriers, herbivore-induced plant volatiles, resistance genes, and non-host resistance mechanisms. This review aims to provide new insights for further research on aphid resistance mechanisms and the genetic basis of resistance in wheat, ultimately supporting efficient and sustainable crop protection.

## 2. Plant Immune Perception and Anti-Defense Strategies Mediated by Aphid Saliva

Aphids are piercing-sucking insects that penetrate the sieve elements of the phloem with their stylets to ingest plant sap, acquiring sugars, amino acids, and other nutrients. During probing and feeding, aphids secrete two types of saliva: gelling saliva and watery saliva. The gelling saliva hardens to form a sheath around the stylets with a protective function. Watery saliva contains complex proteins, including detoxification enzymes, hydrolases, proteases, calcium-binding proteins, and salivary proteins.

There is a complex dynamic interaction between pathogens and host plants, and the “zig-zag model” provides a conceptual framework for understanding this relationship [11]. Some conserved molecules in aphid saliva resemble pathogen-associated molecular patterns (PAMPs) and are recognized by pattern-recognition receptors (PRRs) on the plant cell surface, thereby triggering PAMP-triggered immunity (PTI). Aphids subsequently secrete salivary effectors into the host plant to interfere with PTI, resulting in effector-triggered susceptibility (ETS). However, certain plants have evolved resistance (R) genes encoding proteins capable of recognizing these effectors and activating effector-triggered immunity (ETI), thereby restricting aphid infestation. Aphids modulate plant defense responses by secreting salivary proteins into host tissues through the stylets. These salivary proteins are generally classified into two functional categories: elicitors, which activate plant defense responses, and effectors, which suppress host immunity [7,8]. Recent advances in salivary gland transcriptomics and proteomics have greatly expanded the identification of candidate salivary proteins in wheat aphids, providing an essential foundation for functional characterization of aphid salivary proteins [12,13,14]. Integrative transcriptomic and proteomic analyses have identified 76 and 114 salivary proteins from the watery saliva of *S. avenae* and *S. graminum*, respectively [12,14]. Recently, RNA interference (RNAi) has emerged as a promising strategy for silencing target genes and holds great potential for the research of salivary proteins [15,16]. The use of double-stranded RNA (dsRNA) to specifically silence key genes essential for pest growth and development not only facilitates pest control but also minimizes risks to non-target organisms, particularly the natural enemies of pests. This will facilitate the discovery of key virulence effectors and salivary elicitors, which are of great significance for the breeding and utilization of aphid-resistant wheat varieties.

### 2.1. Wheat Defense Responses Induced by Salivary Elicitors

Plants possess the ability to rapidly and specifically perceive herbivore-derived cues, such as herbivore-associated molecular patterns (HAMPs) and salivary elicitors, which play pivotal roles in activating plant defense responses against herbivore attack [17]. Recognition of these signals typically initiates PTI, leading to a cascade of downstream defense responses. A previous study found that treatment of *A. thaliana* with 3–10 kDa salivary protein fractions from *M. persicae* significantly upregulated the expression of defense-related genes, including *PAD3*, *PR2*, and *WRKY28*, while simultaneously reducing aphid fecundity [18]. Similarly, the symbiont-derived protein GroEL from *M. euphorbiae* was shown to induce PTI response in Arabidopsis, including callose deposition and ROS accumulation, ultimately suppressing aphid performance [19]. The salivary protein Me47 from *M. euphorbiae* induced the expression of PTI-associated genes, including *Lrr22*, *Pti5*, *Gras2*, and *PR1a*, in tomato plants [20]. Moreover, the salivary protein Cathepsin B from *M. persicae* interacts with the phloem-associated protein EDR1, resulting in sustained activation of ROS signaling in vascular tissues. This interaction interferes with aphid feeding behavior and reduces host suitability [21]. Collectively, these findings highlight that aphid salivary elicitors function as key modulators of plant immune signaling.

Advances in functional genomics have greatly facilitated the identification and characterization of aphid salivary effectors in cereal systems. For instance, the Type III secretion system (T3SS) of *Pseudomonas fluorescens* EtAnH has been successfully employed to deliver candidate effectors into wheat leaves for transient expression, providing a powerful platform for functional validation [22]. In wheat, the identification and functional characterization of aphid salivary elicitors are essential for elucidating plant defense mechanisms and facilitating the discovery of resistance-associated genes. Infiltration of watery saliva from *S. avenae* into wheat leaves significantly upregulated the expression of salicylic acid (SA)-related genes, including *PAL* and *PR1*, thereby negatively affecting aphid feeding behavior and development. These findings demonstrate that aphid salivary proteins can directly participate in the induction of wheat defense responses [23]. To date, only two salivary protein elicitors of *S. avenae* have been identified (Table 1). Among them, the small molecular protein GroES, derived from the primary endosymbiont *Buchnera aphidicola* of *S. avenae*, induces hydrogen peroxide (H_2_O_2_) accumulation and callose deposition in wheat, thereby enhancing wheat resistance to aphids [24]. In addition, the salivary protein SmCSP4 from *S. avenae* interacts with the wheat transcription factor *TaWRKY76*, activating SA-mediated defense responses and further strengthening host resistance [25].

### 2.2. Salivary Effector-Mediated Anti-Defense Strategies of Wheat Aphids

During evolution, aphid salivary effectors have acquired critical roles in modulating aphid–plant interactions by suppressing host defense responses and thereby enhancing aphid adaptability. Early functional characterization of aphid salivary effectors was conducted in the pea aphid *A. pisum* [26]. RNAi-mediated silencing of the salivary gland-specific gene *C002* in *A. pisum* impaired phloem feeding and significantly reduced aphid survival and fecundity, demonstrating that C002 is essential for host colonization [27]. Subsequent studies further demonstrated that multiple aphid salivary proteins function as effectors that suppress plant immunity. Transient overexpression of *M. persicae* salivary proteins MpC002, Mp1/PIntO1, and PIntO2, as well as *M. euphorbiae* salivary proteins Me10 and Me23, in tobacco and Arabidopsis significantly increased aphid reproduction, suggesting important roles in suppressing host defense responses [28,29]. Similarly, overexpression of salivary protein Mp55 in *A. thaliana* enhanced aphid performance and host preference, whereas silencing *Mp55* markedly reduced aphid reproduction, confirming its function as a key effector promoting infestation [30]. In addition to directly modulating plant immunity, some aphid effectors function through metabolic interference. For example, the salivary protein diacetyl/L-xylulose reductase (DCXR) from *Aphis craccivora* participates in methylglyoxal detoxification, and its transient expression in pea significantly increased aphid fecundity [31]. Furthermore, previous studies have shown that other important piercing-sucking pests, such as *B. tabaci* and the *Nilaparvata lugens*, also secrete salivary effectors that suppress host plant defense responses [32,33], suggesting that effector-mediated immune suppression represents a conserved strategy among phloem-feeding insects.

Several salivary effectors have subsequently been functionally characterized in wheat aphids (Table 1). The salivary protein Rp1 from *R. padi* suppresses defense responses in barley, thereby facilitating aphid colonization [34]. Likewise, transient expression of the salivary protein Sg2204 from *S. graminum* significantly reduced callose deposition in wheat leaves [12]. Two salivary effectors from *S. avenae*, Sm10 and SmC002, enhance host plant susceptibility and promote aphid growth by suppressing jasmonic acid (JA)-mediated defense responses [35]. In addition, the salivary effector Sm9723 suppresses the expression of defense-related genes associated with both JA and SA signaling pathways, enabling aphids to evade plant immunity [36]. Furthermore, nanocarrier-mediated RNAi has successfully identified several genes that play crucial roles in wheat aphid growth and development, highlighting their potential as RNAi targets for aphid control. For example, silencing the salivary effector genes *Sg2204* in *S. graminum* and *Sm9723* in *S. avenae* effectively suppressed aphid population growth [12,36].

**Table 1:** Salivary elicitors and effectors of wheat aphids.

Salivary Protein	Aphid Species	Functions	References
SmCSP4	*S. avenae*	Activate SA defense pathway	[25]
GroES	*S. avenae*	Trigger defense responses	[24]
Sm10	*S. avenae*	Enhance host plant susceptibility	[35]
SmC002	*S. avenae*	Enhance host plant susceptibility	[35]
Sm9723	*S. avenae*	Suppress plant immunity	[36]
SaCDK	*S. avenae*	Inhibit host defense response	[37]
SaE23	*S. avenae*	Suppress wheat plant defenses	[38]
SaApo AI	*S. avenae*	Inhibit plant hypersensitive response	[39]
Sg2204	*S. graminum*	Suppress wheat defense	[12]
RP1	*R. padi*	Suppress barley defense responses	[34]

**Figure 1:**
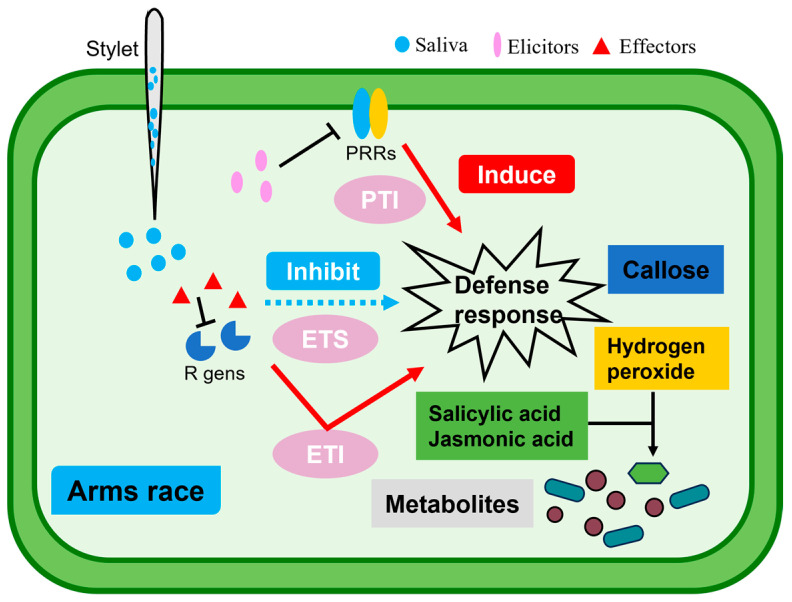
A model depicting the manipulation of plant immunity by aphid salivary proteins.

Figure 1 illustrates how salivary proteins manipulate the immune regulation of plants. Compared with salivary elicitors, salivary effectors are secreted by aphids to suppress or evade host immunity, thereby facilitating successful feeding and reproduction. This represents an evolutionarily acquired anti-defense strategy employed by aphids to overcome plant resistance. Moreover, the outcome of plant–aphid interactions is determined not by individual salivary proteins but by the coordinated action of complex salivary protein mixtures integrating both defense-inducing and defense-suppressing functions. A deeper understanding of the dynamic balance and interplay between salivary elicitors and effectors will be essential for elucidating the molecular mechanisms underlying aphid adaptation and for developing durable strategies for crop resistance.

At present, functional studies on salivary proteins in wheat aphids remain at an early stage, particularly in *S. avenae*, *S. graminum*, and *R. padi*. Notably, these three major wheat aphid species exhibit distinct feeding preferences and damage characteristics on wheat, suggesting that they may employ different salivary effector strategies during infestation. Although several salivary proteins with potential roles in suppressing host immunity, inducing plant defense response, or facilitating phloem feeding have been identified, most studies remain limited to the functional characterization of individual candidate proteins. A systematic understanding of aphid effector repertoires, effector cooperation, and species-specific virulence strategies among wheat aphids is still lacking. Consequently, research on salivary effector-mediated aphid virulence and host adaptation remains far less advanced than that in other plant–insect systems.

## 3. Early Defense Signaling in Wheat Under Aphid Infestation

Plant hormones play an important role in the plant immune response [5]. In response to insect infestation, wheat activates defense pathways mediated by phytohormones such as JA, SA, ethylene (ET), and abscisic acid (ABA), as well as signaling molecules like reactive oxygen species (ROS). These signaling pathways subsequently promote the accumulation of secondary metabolites, thereby affecting aphid growth and development [40]. H_2_O_2_, as a broad-spectrum signaling molecule, accumulates in plant tissues in response to both pest and pathogen infestation [41]. Feeding by *S. avenae* and *S. graminum* leads to a significant increase in H_2_O_2_ content in wheat leaves, with *S. graminum* inducing a stronger accumulation of H_2_O_2_ [42].

The JA and SA pathways are two crucial hormone signaling pathways involved in plant defense against insects. Dynamic interactions between these pathways modulate the levels of defensive metabolites and further influence plant resistance. The SA pathway is reported to primarily mediate plant defense against aphids. For instance, feeding by *S. avenae* induces elevated expression of *pathogenesis-related protein 1* (*PR1*) in the SA signaling pathway of wheat [43]. Transcriptome sequencing and liquid chromatography-tandem mass spectrometry (LC-MS/MS) analyses have also revealed that infestation by both *S. avenae* and *S. graminum* activates an SA-mediated defense response in wheat, accompanied by increased SA accumulation [44]. Previous studies have shown that wheat aphid infestation also induces the expression of JA-related genes and elevates JA levels [45,46]. Spraying wheat seedlings with JA induces the accumulation of defensive compounds in wheat, such as DIMBOA, thereby significantly inhibiting aphid development and fecundity [47]. Although JA and SA were previously considered to act antagonistically, increasing evidence suggests that they can also function synergistically or independently, depending on factors such as plant species [48,49,50]. Additionally, ET enhances plant defense against aphids by promoting cell wall thickening [51]. Feeding by wheat aphids can induce upregulation of the ET pathway marker gene *EIN2* in harpin transgenic wheat lines and suppress aphid performance [52].

## 4. Multi-Layered Wheat Defense Strategies Against Aphids

The perception of aphid attack and the activation of downstream signaling pathways ultimately lead to the deployment of diverse defense responses in wheat. These defense responses represent the functional outputs of plant immunity and play crucial roles in limiting aphid colonization, feeding, and population growth. Following aphid recognition, wheat activates a multilayered defense system that includes structural barriers, the production of defensive metabolites, and indirect defenses mediated by herbivore-induced plant volatiles (HIPVs). Physical defense traits that contribute to resistance against aphids include morphological characteristics, epicuticular waxes, trichomes, and internal structures. These physical barriers constitute the first line of plant defense, interfering with aphid host selection, feeding behavior, growth, and development. Similarly, chemical defense plays a critical role in the plant defense system, involving secondary metabolites, nutrient composition, and other biochemical factors that adversely affect aphid physiology and survival.

### 4.1. Morphological and Physical Defense Against Aphids in Wheat Plants

When aphids migrate into wheat fields in spring, they initially locate host plants using olfactory and visual cues, such as plant color and volatile organic compounds released by wheat plants [53]. Plant coloration serves as an important visual cue influencing aphid host selection. After landing on plant surfaces, tactile cues provide critical information for assessing host suitability for colonization. Morphological traits of wheat, including leaf trichomes, flag leaf characteristics, awn length, and surface wax composition, are key factors influencing aphid colonization. The length and density of leaf trichomes are significantly and positively correlated with wheat resistance to aphids [54]. Leaf trichomes act as a physical barrier to aphid feeding and represent an important component of wheat defense. For example, aphids preferentially feed on older leaves, which generally exhibit lower trichome density [55]. Plant surface wax also plays a crucial role in aphid host selection and feeding behavior. In wheat, leaf wax can inhibit aphid feeding behavior and may exert toxic effects on aphids [56]. Callose is a *β*-1,3-linked glucan that is typically deposited in small amounts at the cell wall or around the sieve pores of phloem sieve plates, and its deposition is closely associated with plant defense against pests and pathogens [57,58]. Feeding by *S. avenae* and *S. graminum* induces substantial callose accumulation in wheat leaf cells [42]. However, the precise mechanism of callose involvement in wheat–aphid interactions remain unclear. It is hypothesized that aphid-induced callose deposition may obstruct the transport of phloem sap to aphid stylets, thereby inhibiting aphid feeding and enhancing wheat resistance.

### 4.2. Chemical Defense Against Aphids in Wheat Plants

#### 4.2.1. Nutritional Components

The nutrients ingested by aphids from wheat leaves significantly influence their growth performance after colonization. Soluble proteins, sugars, amino acids, and inorganic salts in wheat tissues directly affect aphid growth and development, thereby shaping plant resistance to aphid infestation [59]. A previous study reported that the total protein content in the leaves of aphid-resistant wheat varieties was significantly lower than that in susceptible varieties, whereas the concentration of free amino acids in the phloem was higher in resistant varieties [60]. Under high-temperature and drought stress, wheat plants exhibit increased levels of soluble sugars and amino acids, while aphids show reduced net reproductive rates, intrinsic rates of increase, and finite rates of population growth [61]. Both the diversity and composition of amino acids in wheat are associated with resistance to aphids. *β*-aminobutyric acid has been demonstrated to exert an inhibitory effect on wheat aphids. For example, root application of *β*-aminobutyric acid during the seedling stage significantly suppressed aphid weight gain. Moreover, adding *β*-aminobutyric acid into the artificial diet reduced aphid body weight by 55%, indicating a direct toxic effect [62]. However, the relationships between individual amino acids and wheat resistance levels are not always consistent. For instance, the contents of glutamic acid, alanine, lysine, and aspartic acid in wheat leaves are positively correlated with the intrinsic growth rate of aphids, whereas the contents of leucine, isoleucine, valine, and proline are negatively correlated with aphid intrinsic growth rates [63].

#### 4.2.2. Secondary Metabolites

Plant secondary metabolites are products of complex branched metabolic pathways, and many of these compounds contribute to plant resistance against pests and pathogens [5]. A substantial proportion of secondary metabolites function as phytoalexins, thereby strengthening plant defense responses [57]. The major secondary metabolites associated with wheat defense include phenolic compounds, flavonoids, benzoxazinoids (Bxs), and alkaloids.

Among plant secondary metabolites, phenolic compounds constitute one of the most common and widespread groups of defense-related chemicals [59]. Previous studies indicate that phenol levels are positively correlated with plant resistance to insects, as these compounds exert detrimental effects on aphid feeding behavior, growth, and development [64]. The biosynthesis of phenolic compounds is largely regulated by the phenylpropanoid pathway, which plays a crucial role in plant defense. For example, the phenylpropanoid pathway has been shown to contribute to wheat kernel resistance against *Sitodiplosis mosellana* infestation [65]. Lignin, a phenolic heteropolymer, confers resistance to herbivores by forming a mechanical barrier through increased leaf toughness and by reducing leaf nutritional content [66]. The content of *p*-coumaric acid and vanillic acid in wheat seedling leaves was significantly and positively correlated with the resistance level against aphids [67]. Flavonoids, a major subclass of phenolic compounds, play important roles in mediating plant responses to both biotic and abiotic stresses [68]. As cytotoxic secondary metabolites, flavonoids protect plants from insect damage by influencing insect behavior and development [69]. For instance, the flavone tricin in wheat exerted strong antifeedant effects against *S. graminum* [70]. Similarly, supplementation of artificial diets with catechins and quercetin significantly reduced the survival rate of *S. avenae* and *R. padi*, and negatively affected their growth and development [71]. Consistent with these findings, high-performance liquid chromatography (HPLC) analysis revealed a significant positive correlation between quercetin accumulation in wheat kernels and flag leaves and plant resistance to aphids [40].

Bxs represent an important class of secondary metabolites that play a crucial role in chemical defense against a wide range of biotic stresses [72]. Integrative transcriptomic and metabolomic analyses have demonstrated that Bxs function as direct defensive compounds against *Ostrinia furnacalis* in maize [73]. In wheat, Bxs accumulate at higher concentrations in older leaves than in younger leaves, and these compounds are key determinants of wheat resistance to aphids [55]. Among Bxs, hydroxamic acids are considered the most biologically active subgroup [74]. The compound 2,4-dihydroxy-7-methoxy-1,4-benzoxazin-3-one (DIMBOA) is the predominant hydroxamic acid derivative in wheat. Elevated DIMBOA levels are positively correlated with enhanced resistance to *R. padi* and *S. avenae* [75]. DIMBOA functions as a digestive toxin by inhibiting trypsin activity in *Ostrinia nubilalis* larvae [76]. In addition, both DIMBOA and its precursors have been shown to reduce UDP-glucuronosyltransferase activity in *S. avenae* [77]. Furthermore, Bxs content in wheat is altered by aphid feeding, thereby affecting callose deposition and subsequently influencing plant resistance to aphids [78].

#### 4.2.3. Herbivore-Induced Plant Volatiles (HIPVs)

Herbivore attack triggers a wide array of plant defense responses, among which herbivore-induced plant volatiles (HIPVs) represent one of the most rapid and ecologically relevant responses [79]. Volatile compounds released by host plants serve as crucial cues for aphids in locating suitable hosts for settlement. In response to aphid infestation, plants emit complex blends of volatile organic compounds (VOCs), particularly terpenoids, including (*E*)-4,8-dimethyl-1,3,7-nonatriene (DMNT), (E)-*β*-farnesene (EBF), and linalool. HIPVs mediate tritrophic interactions by simultaneously affecting herbivores and their natural enemies. For example, EBF functions both as an alarm pheromone for aphids and as a signaling molecule in plant–insect interactions. It repels aphids while attracting predatory and parasitoid natural enemies, thereby playing an important role in regulating plant–aphid dynamics [80]. Aphid infestation has been shown to induce the emission of multiple VOCs, including 2-camphene, 6-methyl-5-hepten-2-one (MHO), and methyl salicylate (MeSA), as well as increased levels of S-linalool, trans-2-hexenal, and benzaldehyde. Behavioral assays have demonstrated that MHO and MeSA emitted by aphid-resistant wheat varieties exert significant repellent effects on wheat aphids [81]. Similarly, the monoterpene linalool released by octoploid dwarf wheat exerted a repellent effect on wheat aphids [82]. Importantly, field applications of HIPVs such as MeSA and EBF have been shown to repel aphids while simultaneously attracting natural enemies, thereby reducing aphid damage in wheat agroecosystems [83].

**Figure 2:**
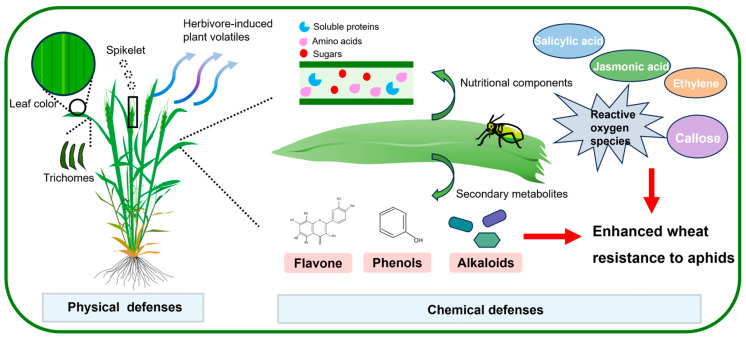
Physical and chemical defenses against aphids in wheat.

Taken together, wheat’s defense against aphids is not mediated by a single mechanism but rather by the coordinated activation of multiple defense layers. Following aphid attack, defense signaling pathways trigger a series of downstream responses, including physical and chemical defenses and HIPV-mediated indirect defenses (Figure 2). These responses reshape aphid host selection, feeding behavior, survival, and fecundity, thereby contributing to wheat resistance. Importantly, the activation of these defense responses is under genetic control, suggesting that resistance genes play a central role in shaping resistance outcomes.

## 5. Genetic Basis of Wheat Resistance

Although the defense mechanisms described above are phenotypic manifestations of resistance, their effectiveness is ultimately determined by underlying genetic factors. Resistance genes represent a direct form of plant defense against herbivorous insects [84]. Molecular breeding for crop resistance to pests and pathogens is considered one of the most economical and effective approaches for crop protection. Molecular mapping of aphid resistance genes facilitates the identification and selection of resistant traits, thereby significantly accelerating the breeding of aphid-resistant wheat varieties [85]. The complex genetic background of wheat, together with its allopolyploid nature, confers high tolerance to genomic modifications. Previous studies have demonstrated that genes conferring resistance to pests, pathogens and abiotic stresses can be successfully introduced into wheat from other cereal crops, including rye, barley and oats [84].

*Dn1* was identified as the first resistance gene against *D. noxia* in wheat. To date, more than 10 resistance genes against *D. noxia* and 15 resistance genes against *S. graminum* have been identified in wheat (Table 2) [84,86]. Currently, the identified resistance genes to *S. avenae* are mainly located on wheat chromosomes 6A and 7D (Table 2). For example, the resistance gene *RA-1*, conferring resistance to *S. avenae* and derived from the durum wheat accession C273, was localized to chromosome 6AL through linkage mapping [87]. In addition, the resistance gene *Sa2*, identified in the Chinese wheat accession XN98-10-35, is on chromosome 7D [85]. In addition, combined analyses of aphid performance and wheat genotypes identified broad-spectrum resistance genes against *S. avenae*, *R. padi*, and *S. graminum* in the wheat lines E12165, Amigo, and Presto triticale, which were mapped to chromosome 1AL.1RSam [88]. By reducing genome complexity using restriction endonucleases, genotyping by sequencing (GBS) provides an efficient platform for gene mapping in wheat. Researchers at the International Maize and Wheat Improvement Center (CIMMYT) used GBS to analyze a recombinant inbred line (RIL) population derived from the aphid-resistant wheat accession CWI76364 and the susceptible accession Seri M82. The analysis identified a quantitative trait locus (QTL) conferring antibiosis resistance to *R. padi* (*QRp.slu.4BL*) on wheat chromosome 4BL, as well as two QTLs associated with tolerance to *R. padi* (*QRp.slu.5AL* and *QRp.slu.5BL*) on chromosomes 5AL and 5BL, respectively [89]. In addition, evaluation of an F_6_ RIL population (n = 226) derived from a cross between the *S. graminum*-resistant line Sokoll and the susceptible line Weebill1 led to the identification of a major resistance locus on chromosome 7DL. Subsequent linkage and QTL analyses enabled the preliminary mapping of the resistance gene, designated *GbSkl* [90].

Although many resistance genes have been identified, the underlying molecular mechanisms remain unclear. The completion of the wheat whole-genome sequencing, together with the emergence of genome-wide association study (GWAS) approaches, has greatly advanced functional genomics research in wheat [109]. A previous study reported that *TaDIR-B1*, a gene associated with resistance to *Fusarium crown* rot in wheat, was first identified using GWAS and QTL mapping [110]. Similarly, GWAS combined with QTL analysis revealed that *TaMTB*, a gene encoding an m^6^A methyltransferase, is significantly associated with resistance to *wheat yellow mosaic virus* (WYMV) [111]. These approaches provide valuable insights for the mapping and functional characterization of aphid resistance genes in wheat. Nevertheless, most studies on the genetic background and linkage mapping of resistance genes remain at a preliminary stage, and limited progress has been made in fine mapping and functional validation.

## 6. Non-Host Resistance: A Promising Source of Durable Aphid Resistance

Unlike host resistance, which is often governed by specific resistance genes and may be overcome by aphid adaptation, non-host resistance (NHR) refers to the resistance exhibited by all genotypes within a plant species against all isolates of a given pathogen [9]. Because NHR is typically mediated by multiple defense layers and coordinated action of numerous genes, it is widely regarded as one of the most durable and broad-spectrum forms of plant resistance [9,10]. Similar to the infection phase of pathogens on non-host plants, aphids attempt to penetrate the leaf surface using their stylets regardless of plant species, suggesting that defense responses in non-host plants are induced at the molecular level [112,113]. To date, studies on plant resistance to insects have focused predominantly on the mechanisms underlying compatible plant–insect interactions. Therefore, understanding the mechanisms underlying NHR may provide valuable resources for the development of durable aphid-resistant wheat cultivars.

### 6.1. The Molecular Framework Associated with Non-Host Resistance Against Insects

NHR reflects the overall effectiveness of plant defense systems against diverse biotic challenges and should be regarded as a phenomenon arising from the collective action of multiple defense layers, rather than as a distinct branch of plant immune pathways attributable to a single mechanism [114]. Similar to host resistance, NHR involves aphid recognition, activation of defense signaling pathways and defense proteins. However, these responses are often stronger, more rapid, and more effective in non-host interactions, thereby preventing successful aphid colonization.

Aphid non-host resistance begins with aphids being unable to successfully locate, penetrate or utilize host tissues. Aphid stylet penetration of plant tissue is well characterized using the electrical penetration graph (EPG), which can distinguish different types of plant–aphid interactions. For example, in the Arabidopsis-*R. padi* (non-host) interaction, aphid stylets fail to reach the phloem for feeding [115]. However, in the barley-*M. persicae* (poor-host) interaction, the aphid can successfully penetrate sieve-tube elements and ingest phloem sap, but the level of ingestion is reduced compared with that in host interactions, suggesting that resistance in different interaction types likely occurs at distinct plant cell layers [115,116]. Aphid salivary components are considered signaling molecules that trigger defense activation in non-host plants [7,27]. Silencing the tomato gene *TFT7*, which interacts with the salivary protein Me10 of *M. euphorbiae*, has no effect on host susceptibility to the potato aphid but enhances the survival and fecundity of the non-host aphid *Aphis gossypii* [117]. These findings suggest that NHR can operate at multiple stages of aphid feeding and may involve distinct defense barriers in different plant tissues.

Following aphid probing, non-host plants activate defense signaling pathways similar to those involved in host resistance. Among the signaling pathways implicated in NHR, JA is a key regulatory factor of NHR. For example, suppression of JA-dependent defense enabled colonization by previously non- adapted herbivore species capable of successful feeding and reproduction, highlighting the critical role of JA in shaping herbivore host selection [118]. When the poor-host plant wheat was fed by *M. persicae*, a strong immune response was activated, with differentially expressed genes (DEGs) significantly enriched in alpha-linolenic acid metabolism and accompanied by a marked increase in JA accumulation [119]. The activation of defense signaling pathways ultimately leads to the accumulation of defense-related proteins that restrict aphid performance. For instance, transcriptional analyses in barley revealed that infestation by *M. persicae* (a non-host interaction) triggered stronger defense responses, and several thionins were found to contribute to aphid resistance [120]. Similarly, transcriptomic analyses of the Arabidopsis-*R. padi* non-host interaction identified multiple DEGs associated with aphid resistance, including the toxic protein-related gene *Vegetative Storage Protein 1* (*VSP1*), which contributed to non-host resistance against aphids [121]. In addition, Bowman–Birk trypsin inhibitor (*TaBBTI-1*), specifically induced during *M. persicae* infestation on wheat (a poor-host interaction), significantly suppressed the fecundity of both *S. avenae* and *M. persicae*, as well as the feeding capacity of *Helicoverpa armigera*, demonstrating broad-spectrum resistance against herbivorous insects [119]. Collectively, these findings suggest that defense proteins represent important downstream components of aphid NHR.

### 6.2. Exploiting NHR for Durable Wheat Resistance

Taken together, current evidence suggests that aphid non-host resistance is not mediated by a single defense mechanism but rather by the coordinated action of multiple defense layers. Notably, many of these mechanisms overlap with those involved in host resistance, but they appear to be activated more effectively during non-host interactions. The widespread adoption of genome editing and omics approaches has provided a strong foundation for the precise and efficient utilization of NHR-associated traits in agriculture. Exploiting non-host resistance genes represents a promising strategy for breeding crops with broad-spectrum and durable resistance. Genes involved in NHR have already been successfully applied in plant breeding to confer resistance against pathogens [122,123]. Given the mechanistic similarities between aphid–plant and pathogen–plant interactions, NHR is increasingly regarded as a promising strategy for developing broad-spectrum and durable aphid-resistant crops [124].

## 7. Conclusions and Perspectives

Current evidence indicates that wheat resistance to aphids is governed by a multilayered defense system involving aphid recognition, immune signaling, physical and chemical defenses, resistance genes, and non-host resistance mechanisms. Aphid salivary proteins play an important role in wheat–aphid interaction, acting either as elicitors that activate plant defenses or as effectors that suppress host immunity. Importantly, resistance is not determined by a single defense component but by the coordinated action of multiple defense layers that collectively influence aphid performance and host suitability. Despite substantial progress, several major bottlenecks continue to hinder the development of durable aphid-resistant wheat. Firstly, only a limited number of aphid salivary effectors have been functionally characterized, and their molecular targets in wheat remain largely unknown. Secondly, the regulatory networks linking aphid perception to downstream defense responses are still poorly understood. Thirdly, relatively few resistance genes have been effectively utilized in breeding programs, and resistance conferred by individual genes may be overcome by aphid adaptation. Finally, the mechanisms underlying non-host resistance remain insufficiently explored despite their considerable potential for durable resistance breeding.

Future research should move beyond conventional germplasm screening to uncover the core biological mechanisms underlying durable aphid resistance. Priority should be given to the combinatorial action of salivary effectors, rather than single effector proteins. It is likely that aphid salivary proteins function cooperatively to manipulate host plant immunity and hormone signaling. Therefore, future studies should aim to construct “aphid salivary effector networks” through integrated transcriptomics, proteomics, and protein–protein interaction analyses, combined with functional validation in wheat. In parallel, elucidating the molecular basis of NHR may reveal novel defense mechanisms and resistance-associated genes that can be incorporated into wheat breeding programs. The integration of multi-omics approaches, spatial transcriptomics, genome-wide association studies, and functional genomics tools such as RNAi and CRISPR-based genome editing will greatly accelerate the discovery and validation of resistance determinants. Ultimately, combining host resistance genes, endogenous defense pathways, and NHR mechanisms may provide a promising strategy for developing wheat cultivars with durable and broad-spectrum resistance to aphids. Such advances will contribute to environmentally sustainable aphid management and global food security.

## Figures and Tables

**Table 2:** Wheat aphid resistance genes.

Resistance Genes	Chromosome	Aphid Species	Crops	References
*RA-1*	6AL	*S. avenae*	*T. durum*	[87]
*Sa2*	7D	*S. avenae*	*T. aestivum*	[85]
*Gb1*	-	*S. graminum*	*T. aestivum*	[91]
*Gb2*, *Gb6*	1A	*S. graminum*	*S. cereale*	[92,93]
*Gb5*	7AL	*S. graminum*	*Ae. speltoides*	[94]
*Gb3*, *Gb4*, *Gb7*, *Gb8*, *Gb9*, *Gba*, *Gbb*, *Gbc*, *Gbd*, *Gbx1*, *Gbz*	7DL	*S. graminum*	*Ae. tauschii*	[95,96,97,98,99,100]
*SgR1*	-	*S. graminum*	*S. bicolor*	[101]
*Dn1*, *Dn2*, *Dn5*, *Dn6*, *Dn8*, *Dnx*, *Dn2401*, *Dn626580*, *Dn100695*	7DS	*D. noxia*	*Ae. tauschii*	[86,102,103,104,105,106,107]
*Dn7*	1B	*D. noxia*	*S. cereale*	[108]

## Data Availability

No new data were created or analyzed in this study.
